# Epigenetic signatures on plasma cell-free DNA to detect kidney allograft rejection in a non-invasive way: development of a 10-plex digital PCR assay

**DOI:** 10.1186/s40364-025-00834-7

**Published:** 2025-09-26

**Authors:** Yoan Velut, Geoffroy Poulet, Thomas Bersez, Pauline Boyer, Coraline Maujean, Stéphanie Gnanalingam, Camille Moniot, Jana Heneine, Dany Anglicheau, Marion Rabant, Renaud Snanoudj, Vincent Vuiblet, Gabriel Choukroun, Tristan de Nattes, François Audenet, Bastien Parier, Sophie Ferlicot, Virginie Verkarre, David Buob, Pierre Galichon

**Affiliations:** 1 CGenetix, Company, Head office, 7 rue de Laborde, Paris, 75008 France; 2 Department of Nephrology and Kidney Transplantation, Necker Hospital, AP-HP, and Université Paris Cité, Necker-Enfants Malades Institute, INSERM U1151, CNRS UMR8253, Paris, France; 3 Université Paris Cité, Inserm U1151, Institut Necker-Enfants Malades Pathology department, Hôpital Necker-Enfants Malades, AP-HP, Paris, France; 4 Kremlin-Bicêtre, AP-HP, Villejuif, France; 5 CHU REIMS, Reims, France; 6 CHU Amiens-Picardie, Amiens, France; 7 Univ Rouen Normandie, INSERM U1234, CHU Rouen, CIC-CRB 1404, Nephrology Department, Rouen, F-76000 France; 8 Department of Urology, Hôpital Européen Georges Pompidou, AP-HP.Centre, Université Paris Cité, Paris, France; 9 HEGP AP-HP, Paris, France; 10 Tenon AP-HP, Paris, France; 11 Sorbonne Université, INSERM UMR_S1155 CoRaKID Unit, AP-HP, APHP.Sorbonne Université, Paris, France

**Keywords:** Kidney allograft, Rejection, Cell-free DNA, Epigenetic, digital-PCR

## Abstract

The standard of care for the follow-up of kidney allograft recipients combines non-invasive but non-specific biomarkers and kidney biopsies for the gold standard histology-based diagnosis, limited by the sampling bias, haemorrhagic risk, and low cost-effectiveness. We hypothesized that a targeted epigenetic analysis of cell-free DNA (cfDNA) would combine non-invasiveness and specificity for the diagnosis of kidney allograft rejection. We developed an in silico pipeline to identify 9 specific methylation signatures of epithelial or endothelial cell types in glomerular and tubular kidney compartments. Methylation-specific digital Polymerase Chain Reaction (dPCR) were designed and validated for these markers and combined in a 10-plex dPCR. In a retrospective cohort of 170 plasma cfDNA from adult kidney transplant recipients, we evaluated the diagnostic properties of our biomarkers for predicting rejection, evaluated on solid biopsy according to Banff 2022 classification. Combining the dedicated biomarkers with standard-of-care blood tests (donor-specific antibody (DSA), estimated glomerular filtration rate (eGFR)) produced a prediction model with an Area under the Curve (AUC) for biopsy-proven kidney transplant rejection vs. no rejection greater than with DSA and eGFR alone (AUC = of 0.884 vs. 0.776, *p* = 0.0005). In an alternative model for the prediction of any graft lesion of Banff classification vs. pristine biopsies (all Banff score = 0) epigenetic kidney biomarkers outperformed DSA (AUC = 0.754 vs. 0.596, *p* = 0.004). Thus, epigenetic signatures derived from the combination of kidney cell type specific methylation marker of cfDNA constitute a promising non-invasive diagnostic and theragnostic tool for kidney transplant patients.

**Supplementary Information:**

The online version contains supplementary material available at 10.1186/s40364-025-00834-7.

To the Editor,

Rejection is a cause of kidney allograft failure limiting the benefits of the best treatment for end-stage kidney disease [1]. Screening tests for rejection in blood (creatinine, donor specific antibodies (DSA), donor-derived cell free DNA (cfDNA)) or urine (proteinuria and chemokines) are non-specific and require biopsy for confirmation [2–5]. cfDNA methylation to quantify organ or cell-specific injuries is promising, notably in cancer, but has not been studied in kidney transplantation [6–8]. Here, we discover, develop and validate a set of kidney-specific cfDNA methylation marks quantified by digital PCR, and determine its diagnostic performance for predicting kidney allograft biopsy results.

## Kidney-specific methylation marks in humans

**Fig. 1:**
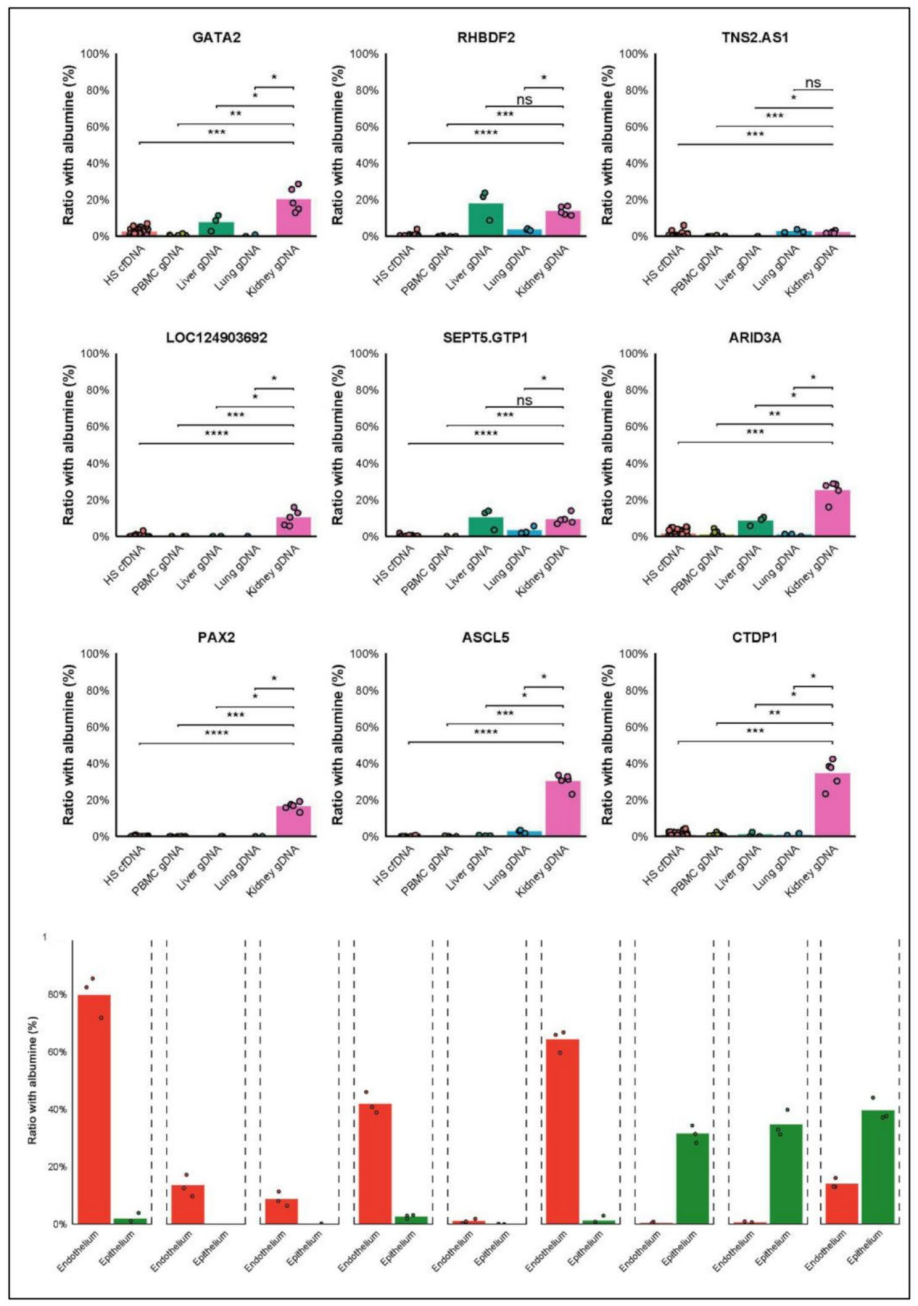
Organ and cell-type specificity of methylation marks. Relative quantification of each epigenetic kidney biomarker in synthetic and tissue control samples (Top). In control sample of tissue and Peripheral blood mononuclear cells. *n* = 50 plasma-cfDNA samples from healthy subjects (red), *n* = 10 gDNA from Peripheral blood mononuclear cells isolated from healthy patient blood, *n* = 3 gDNA from liver and lung and *n* = 5 gDNA from kidney qualified by a certified pathologist as healthy peritumoral tissues. Relative quantification of each epigenetic kidney biomarker on kidney endothelial and epithelial cell gDNA (Down). Each biomarker is normalized relative to the number of genomes in each sample represented by the internal control Albumin gene. *n* = 3 gDNAs from Epithelial cell adhesion molecule positive kidney tubular epithelial cells fraction, *n* = 3 gDNAs from CD105 (+) kidney tubular endothelial cells fraction

We reanalyzed publicly available methylome data of 21 human tissues and cell types of interest in triplicate from Gene Expression Omnibus (GSE186458) [9]. From 15,678,715CpG islands available in ≥ 2 replicates for 21 anatomical regions, we identified specific methylation of whole kidney and renal subcompartments (tubular/glomerular epithelial cells, podocytes, peritubular/glomerular endothelial cells) vs. all other anatomical regions (Fig. 1). 9 kidney-specific biomarkers was selected, with special attention given to their negativity in blood cells, the main contaminants of plasma cfDNA with cellular DNA.

We selected “CTDP1” for both vascular and epithelial renal fractions, “ARID3A”, “GATA2”, “LOC124903692”, “RHBDF2”, “SEPT5-GP1BB” and “TNS2-AS1” for the renal endothelial fraction, “PAX2” and “ACSL5” for the renal epithelial fraction.

## Simultaneous quantification of a kidney-specific panel of CfDNA methylation markers by 10plex dPCR

Primer and probes design, concentrations and coupling were designed for multiplex quantification of the biomarkers, with albumin as internal control. High methylation rate (58 to 98%) in methylated controls, and none in unmethylated controls validated the assay. dPCR was reproducible (mean standard deviation ≤ 0.05%) and sensitive on serial dilutions (detection limit of 1 to 4 copies after bisulfite conversion causing 67 to 80% DNA loss from a minimal input of 0.03 ng = 9–10 DNA copies per biomarker) (Supplementary_Data_2). Quantification of genomic DNA (gDNA) from healthy human organs was significantly higher in the kidney compared to other samples. Detection of our biomarkers in blood cells gDNA and healthy subject cfDNA was minimal, supporting its use for detecting organ injury. Methylation was also found in the lung for TNS2.AS1 and in the liver for SEPT5.GTP1 and RHBDF2, respectively, suggesting that deconvolution might improve kidney-specific quantifications in multiple organ injuries [6]. Furthermore, we studied gDNA from flow cytometry-isolated cell types to evaluate the cell-specificity of our markers within the kidney. Endothelial and epithelial biomarkers were enriched respectively in endothelial and epithelial cells. Low albeit specific relative amounts of SEPT5.GTP1 and TNS2.AS1 (< 10% ratio with albumin) in sorted endothelial cells is relevant to the reported variety of endothelial cells in the kidney. On the opposite, CTDP1 qualified as a pan-renal marker found in both renal epithelial and endothelial gDNA sample, as predicted in silico. Together, these results verify the kidney-specific nature of our candidate biomarkers.

## Diagnostic performances of the CfDNA methylation signature for the diagnosis of kidney allograft rejection

**Fig. 2:**
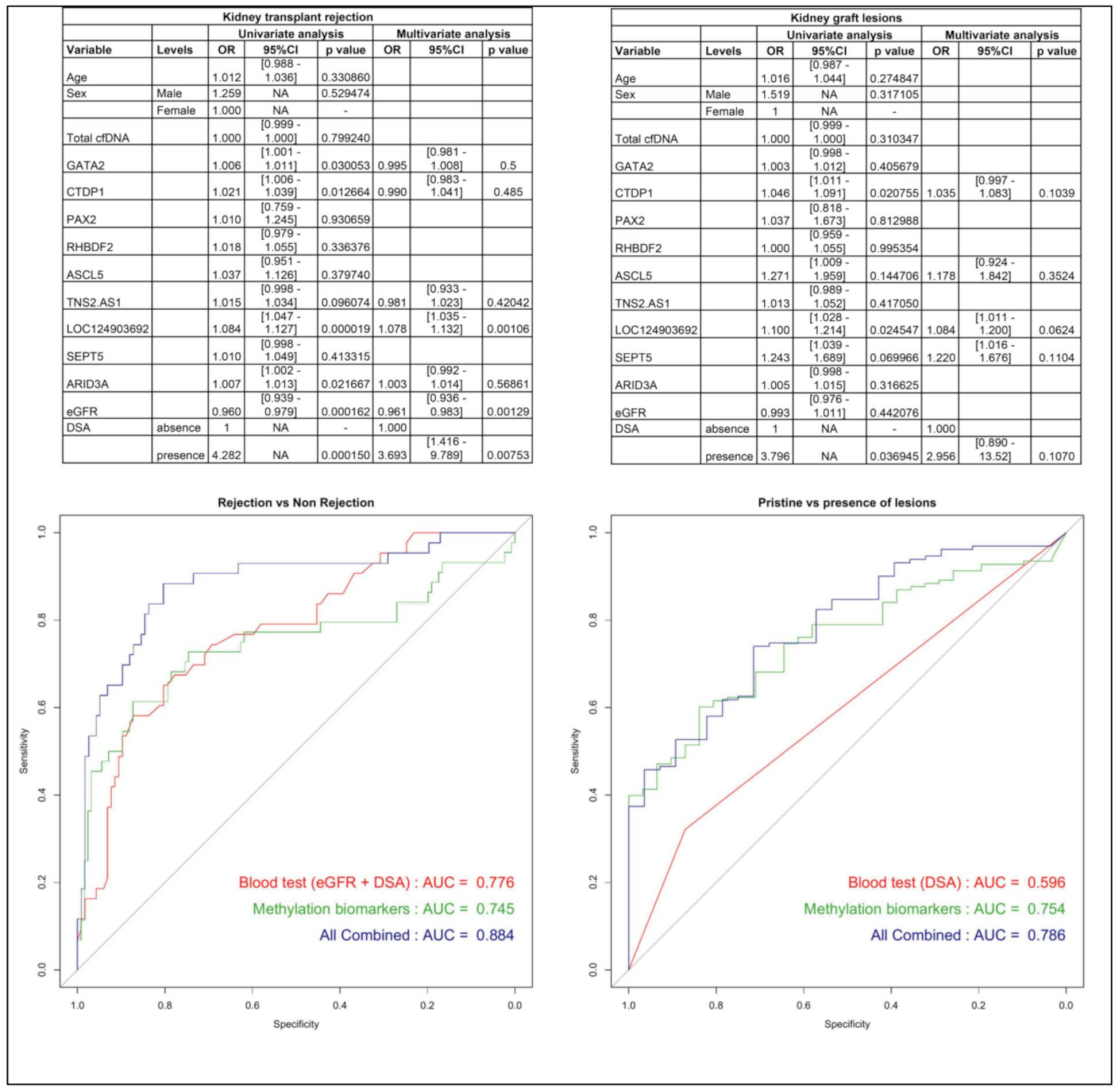
Predictive value of cfDNA methylation markers for kidney injury, compared to classical biomarkers. Univariate and multivariate analysis for kidney transplant rejection or any Banff lesion on biopsy (Up). OR: odds ratio. CI : Confidence Interval ROC curves of prediction models for presence of any Banff lesion on biopsy (Down). DSA: Donor specific antibody. eGFR: estimated Glomerular Filtration Rate

We performed our 10plex dPCR in 170 plasmas collected from EDTA or PAXgene tubes before kidney allograft biopsy. 44 biopsies showed rejection (34/89 indication biopsies, 10/76 surveillance biopsies – 24 Antibody-mediated rejection, 13 T-cell mediated rejection and 7 mixed rejection) and associated in univariate analysis with eGFR & DSA, but also with methylation markers for the whole kidney (CTDP1) and renal endothelial cells (LOC124903692, ARID3A, TNS2.AS1, GATA2). In multivariate analysis, eGFR (*p* = 0.0013), DSA (*p* = 0.0075) and LOC124903692 (*p* = 0.00106) remained significantly associated with rejection (Fig. 2). A model combining eGFR, DSA and an epigenetic signature (derived from a regression model including methylation marks significantly associated with rejection) predicted rejection better than eGFR + DSA or methylation markers alone (AUC = 0.884 vs. 0.776 & 0.745 respectively), suggesting that it could detect the pathogenicity of DSA [10]. In another multivariate analysis, the presence of any lesion (not limited to rejection) was associated with DSA (*p* = 0.1077), LOC124903692 (*p* = 0.0624), another kidney endothelial marker (SEPT5, *p* = 0.1104) and the whole kidney marker CTDP1 (*p* = 0.1039). The presence of any Banff lesion was predicted by methylation markers (AUC = 0.754), whereas DSA performed poorly (AUC = 0.596). These results suggest that the methylation biomarkers are associated with a variety of kidney injuries including antibody-mediated rejection. Thus, quantification of methylated cfDNA is a promising non-invasive technique to improve the prediction of histological kidney injury, needing validation in prospective studies.

## Supplementary Information

Below is the link to the electronic supplementary material.

Supplementary Material 1

Supplementary Material 2

Supplementary Material 3

## Data Availability

The dataset used and/or analysed during the current study are available in the GEO repository (GSE186458): https://www.ncbi.nlm.nih.gov/geo/query/acc.cgi?acc=GSE186458 as published by Loyfer, N., Magenheim, J., Peretz, A. et al. A DNA methylation atlas of normal human cell types. Nature 613, 355–364 (2023). 10.1038/s41586-022-05580-6.

## Abbreviations

AUC: Area under the Curve
cfDNA: Cell-free DNA
dPCR: Digital-PCR
DSA: Donor-specific antibodies
eGFR: Estimated glomerular filtration rate
gDNA: Genomic DNA

## References

[1] Sablik M, Sannier A, Raynaud M, Goutaudier V, Divard G, Astor BC et al. Microvascular Inflammation of Kidney Allografts and Clinical Outcomes. N Engl J Med. 20 févr. 2025;392(8):763–76. 10.1056/NEJMoa2408835 39450752

[2] Roufosse C, Simmonds N, Clahsen-van Groningen M, Haas M, Henriksen KJ, Horsfield C, et al. A 2018 reference guide to the Banff classification of renal allograft pathology. Transplantation. 2018;102(11):1795–814. 30028786 10.1097/TP.0000000000002366 PMC7597974

[3] Gupta G, Athreya A, Kataria A. Biomarkers in kidney transplantation: A rapidly evolving landscape. Transplantation 1 Mars. 2025;109(3):418–27. 10.1097/TP.0000000000005122 39020463

[4] Morgan TA, Chandran S, Burger IM, Zhang CA, Goldstein RB. Complications of Ultrasound-Guided renal transplant biopsies. Am J Transpl Off J Am Soc Transpl Am Soc Transpl Surg Avr. 2016;16(4):1298–305. 10.1111/ajt.13622 26601796

[5] Jiménez-Coll V, Band EKE, Llorente J, González-López S, Fernández-González R, Martínez-Banaclocha M et al. H, All That Glitters in cfDNA Analysis Is Not Gold or Its Utility Is Completely Established Due to Graft Damage: A Critical Review in the Field of Transplantation. Diagn Basel Switz. 6 juin. 2023;13(12):1982. 10.3390/diagnostics13121982 PMC10297394 37370877

[6] Moss J, Magenheim J, Neiman D, Zemmour H, Loyfer N, Korach A, et al. Comprehensive human cell-type methylation atlas reveals origins of Circulating cell-free DNA in health and disease. Nat Commun 29 Nov. 2018;9(1):5068. 10.1038/s41467-018-07466-6 PMC6265251 30498206

[7] Benhaim L, Bouché O, Normand C, Didelot A, Mulot C, Le Corre D, et al. Circulating tumor DNA is a prognostic marker of tumor recurrence in stage II and III colorectal cancer: multicentric, prospective cohort study (ALGECOLS). Eur J Cancer Oxf Engl 1990 Déc. 2021;159:24–33. 10.1016/j.ejca.2021.09.004 34731746

[8] Beinse G, Borghese B, Métairie M, Just PA, Poulet G, Garinet S, et al. Highly specific Droplet-Digital PCR detection of universally methylated Circulating tumor DNA in endometrial carcinoma. Clin Chem 1 Juin. 2022;68(6):782–93. 10.1093/clinchem/hvac020 35323926

[9] Loyfer N, Magenheim J, Peretz A, Cann G, Bredno J, Klochendler A, et al. A DNA methylation atlas of normal human cell types. Nat Janv. 2023;613(7943):355–64. 10.1038/s41586-022-05580-6 PMC9811898 36599988

[10] Parajuli S, Joachim E, Alagusundaramoorthy S, Aziz F, Blazel J, Garg N, et al. Donor-Specific antibodies in the absence of rejection are not a risk factor for allograft failure. Kidney Int Rep Août. 2019;4(8):1057–65. 10.1016/j.ekir.2019.04.011 PMC6698321 31440696

