# Supplementary material for: Epigenetic signatures on plasma cell-free DNA to detect kidney allograft rejection in a non-invasive way: development of a 10-plex digital PCR assay
Source: Biomark Res. 2025 Sep 26;13:118. doi: 10.1186/s40364-025-00834-7 (PMC12465476; doi:10.1186/s40364-025-00834-7)
Supplement: Supplementary file 3 — Supplementary Material 3 [file 40364_2025_834_MOESM3_ESM.docx]

**Methods**

**Generation of core collection of Whole Genome Bisulfite Sequencing of multiple organ**

Publicly available methylation data from multiple human tissues and cell types (Loyfer et al.) were downloaded from the Gene Expression Omnibus (GEO) website. BED graph data was aggregated to create methylation reference profiles for 21 different tissues and cell types of interest, with 3 to 22 sequencing replicates per tissue and cell type (see Supplementary Data). The sequencing replicates were merged and averaged to retain CG positions covered in at least two replicates. CG positions with a standard deviation β value greater than 0.25 across all replicates were filtered out. CpG coverage and redundancy per core collection are shown in Supplementary Figure 1 and Supplementary Figure 2 respectively.

**In silico renal epigenetic biomarker discovery**

Candidate methylation biomarkers were determined based on β coefficient value, defined as the ratio of methylated sequencing reads to total reads (methylated + unmethylated) at each CpG site (0=unmethylated and 1=fully methylated). For the 5 renal cell types of interest (glomerular endothelium, peritubular endothelium, podocytes, kidney glomerular total epithelium and tubular epithelial cells) positions with a β value greater than or equal to 0.5 were identified. Among those positions, ones with a methylation value less than or equal to 0.3 in the 15 other cell types and tissues were retained. To further narrow down the list of candidates, methylation within a 120-base-pair window around each candidate was inspected. Specifically, average β values, β value standard deviations, and total number of CG sites were computed. A detailed output example is shown in Supplemental Figure 3.

Candidate biomarkers were manually inspected to identify those with the best discrimination between the cell type of interest and the others. High differences in methylation between the cell type/tissue of interest and the other reference profiles, as well as the number of CGs surrounding the candidates, were retained as the key sorting criteria. We selected 9 biomarkers: “GATA2”, “CTDP1”, “PAX2”, “RHBDF2”, “SEPT5-GP1BB”, “ARID3A”, “TNS2-AS1”, “ASCL5”, “LOC124903692” with their respective heatmaps shown in Supplementary Table 1.

**Organ and cell sample collection for biomarker development and validation**

A collection of DNA from human tissue was set up. 8µm thick, Formalin-Fixed Paraffin Embedded (FFPE) tissue section of kidney (n=5), liver (n=3), bladder (n=3), heart (n=3), brain (n=3), colon (n=3), stomach (n=3), pancreas (n=3), qualified as “healthy” by a pathologist, have been collected from the Centre des Ressources Biologiques of Hospices Civils de Lyon (BB-0033-00046).

Blood samples from Healthy subjects were purchased at Etablissement Français du sang (site DECINES, 69150, France and site PARIS, 75008). n=30 Healthy donor plasma samples were generated by collecting blood in Paxgene cell-free DNA tubes (Becton Dickinson, ref: 768165) followed by 10min centrifugation at 1200g at room temperature and plasma collection. n = 10 Peripheral blood mononuclear cells (PBMCs) samples were generated by collecting blood in Dipotassium Ethylène Diamine Tétra-Acétique (K2-EDTA) tubes and centrifugation on Ficoll-Paque Plus (Merck) according to manufacturer’s instructions.

**Kidney dissociation, renal cell isolation and cell sorting**

Fresh human kidney cortex tissue samples have been collected at the time of surgery from total nephrectomy. Samples have been qualified as healthy cortex kidney by an approved pathologist and stored in HypoThermosol™ FRS (STEMCELL Technologies) for transfer to the laboratory at 4°C. Patient were informed of sample collection and filed a written consent before surgery. Collection of healthy kidney tissue was approved in compliance with the article L.1121-1 of French law.

Samples were dissociated from an adaptation of a protocol published by Loyfer *et al.* (9). Cell suspension was enriched in endothelial cells by positive enrichment of endothelial cells using mouse anti-human CD105 magnetic beads (Miltenyi). Epithelial and endothelial cells enriched suspensions were stained separately in PBS + 0.5% BSA with specific panel of fluorescent-labeled antibody 30 min in ice, washed twice in PBS + 0.5% BSA and then sorted by FACS on FACS Aria II (Becton Dickinson). Complete list of antibodies, corresponding sorted cell population and gating strategy (made on FlowJo^TM^ v10) are shown in Supplementary Table 2, Supplementary Table 3, Supplementary Figure 4 and Supplementary Figure 5. Sorted cells were centrifuged 5min at 500g, then the supernatant was removed, and cell pellets were stored at -20°C.

**Kidney transplant patients (KTRs) patient recruitment for validation of the clinical relevance of the biomarkers**

To validate the clinical relevance of the epigenetic biomarkers, a retrospective multicentric study was set up by gathering blood samples collected on K2-EDTA tubes (FisherScientific) or Paxgene cell-free DNA tubes (Beckson Dickinson) from adult KTRs eligible for an indication biopsy as part of routine care from the Centre des Ressources Biologiques of French hospitals. A total of N= 181 blood samples were eligible (N=81 from Hôpital Tenon AP-HP, N=52 from Centre Hospitalier Universitaire (CHU) de Reims, N=36 from Hôpital du Kremlin-Bicêtre - Assistance Public-Hôpitaux de Paris (AP-HP), N=12 from CHU Amiens-Picardie). 11 samples were excluded due to incomplete clinical data (see Supplementary Figure 6). The study received the ethical approval from the dedicated French ethic committees and the patients provided written informed consent. Clinical characteristics of the cohort are shown in Supplementary Table 4.

1 to 3 mL of plasma was prepared within 4h storage at RT after blood collection for K2-EDTA (n=139) and in the 10 days for Paxgene cfDNA (n=31) by centrifugation of blood tubes at low speed (1200g, 10 min at RT), then at high-speed (8000g, 10 min at RT). Samples were stored in the Centre des Ressources Biologiques at -80°C until transfer to the laboratory.

The adjudication of diagnoses of rejection was performed automatically from the elementary lesion score of the Banff classification, following the method of Yoo et al, Nature Medicine 2023 (DOI: [10.1038/s41591-023-02323-6](https://doi-org.proxy.insermbiblio.inist.fr/10.1038/s41591-023-02323-6)).

**DNA extraction and conversion**

For genomic DNA extraction, QIAamp DNA FFPE Advanced Kit (Qiagen) was used for FFPE samples and QIAamp DNA Micro Kit (Qiagen) for PBMC from healthy subjects and FACS sorted cells according to manufacturer’s instructions.

Plasma cfDNAs from healthy subjects and KTRs patients have been extracted on EZ2 Connect automation (Qiagen) using the EZ1&2 ccfDNA Kit (Qiagen) according to manufacturer’s instructions. DNA was eluted in 45 µL volume and stored at - 20°C until further notice.

Bisulfite conversion of DNA was used to transform epigenetic information into a genomic footprint using EZ DNA Methylation-Lightning Kit (Zymo Research) according to manufacturer’s instructions. For every bisulfited sample, elution volume post-conversion used was 11 mL.

**10-plex dPCR analysis**

10-plex digital-PCR assay has been designed for simultaneous quantification of each 9 biomarkers candidates and “A” sequence in the same reactive well. A set of primers & TaqMan probes (Eurogentec) have been developed for every candidate biomarker from *in silico* analysis (Supplementary Table 5). Sequences of primers and TaqMan probes targeting each candidate can be found in Supplementary Table 6.

Between 1 to 10 ng of converted DNA was input into digital-PCR wells (Ruby Chip, Ref: C16011, Stilla Technologies) with the digital-PCR reactive mix (10X naica® PCR MIX - 0,750 mL, Ref : R10106 , Stilla Technologies), and specific primers and probes to reach a final reaction volume of 5.5 µL. Assay mix containing primers and probes is detailed in Supplementary Table 5.

Droplet Generation and PCR amplification were carried out on Nio digital-PCR System (STILLA technologies). The PCR program started with 3’ of hold at 95 °C followed by 45” of denaturation at 94 °C and 30’ of combined annealing and elongation step at 58,2 °C. These steps were followed for 45 cycles. Nio Reader Software (STILLA technologies) was used to quantify each candidate in copy/µL of input, and then converted into copy/mL of plasma sample according to the DNA input in the respective analytical steps using the following formula:

$$[cp/ mL plasma] = [cp/ \mu L ]*\left( \frac{11}{9} \right)*\left( Elution bisulfite \left( uL \right) \right)*\frac{Elution of extraction (uL)}{Input bisulfite. \left( uL \right)*Input plasma(mL)}$$

where [cp/mL plasma] is the copy number of DNA molecules into plasma, [cp/µL] is the copy number of DNA molecules counted in the digital-PCR well reaction, [Elution bisulfite] is the elution volume used after bisulfite conversion, [Input bisulfite] is the volume of sample input into the bisulfite conversion column, [Elution of extraction] is the elution volume for cfDNA extraction, [Input plasma] is the volume of plasma used at the beginning of the cfDNA extraction and 11/9 is a dilution coefficient specific to the instrument and chips used.

Relative quantification of biomarkers compared to the total copy number of genome, represented by “Albumin”, was calculated by dividing the copy number of a given biomarker by the corresponding copy number of “Albumin” in a given sample.

Synthetic unmethylated Human Genomic DNA (Zymo Research) and fully methylated Human Genomic DNA (Zymo Research) were used as negative and positive control respectively for biomarker optimization of quantification. Relative quantification of each kidney biomarker in synthetic controls is shown in Supplementary Figure 7. Mean quantification of kidney biomarkers in the multicentric cohort of N=170 KTRs are shown in Supplementary Table 7.

**Analytical sensitivity**

Analytical sensitivity of the assay for each marker was determined by multiple dilution of synthetic unmethylated Human Genomic DNA with fully methylated Human Genomic DNA in ultrapure water. 6 dilutions of fully methylated Human Genomic DNA were performed for a final quantity of 10ng of total DNA (10ng of methylated DNA mixed with 0 ng non-methylated DNA ; 3ng of methylated DNA mixed with 7ng non-methylated DNA; 1ng of methylated DNA mixed with 9ng non-methylated DNA; 0.3ng of methylated DNA mixed with 9.7ng non-methylated DNA; 0.1ng of methylated DNA mixed with 9.9ng non-methylated DNA; 0.03ng of methylated DNA mixed with 9.97ng non-methylated DNA) (see Supplementary Data 2). Each sample were performed in triplicate by 3 different operators and 3 different time. Sample were bisulfite converted and analyzed by dPCR as previously shown (Supplementary Figure 8).

**Statistical analysis**

Statistical analysis was performed using R software (version 4.0.2). Generalized linear models and Area under the Curve (AUC) were computed using *pROC* package (version 1.18.5). Significant factors (p<0.15) in the univariate analysis were selected for the multivariate analysis. For the regression model comparing pristine vs presence of any lesions, pristine biopsy is defined as Banff classification score of 0 for each item (g,i,t,v,ptc,cg,ci,cv,ct,ah,mm). Biopsy is defined “with lesions” if at least one of the items is equal or superior to 1. Unpaired Wilcoxon test was used to compare two groups.

**
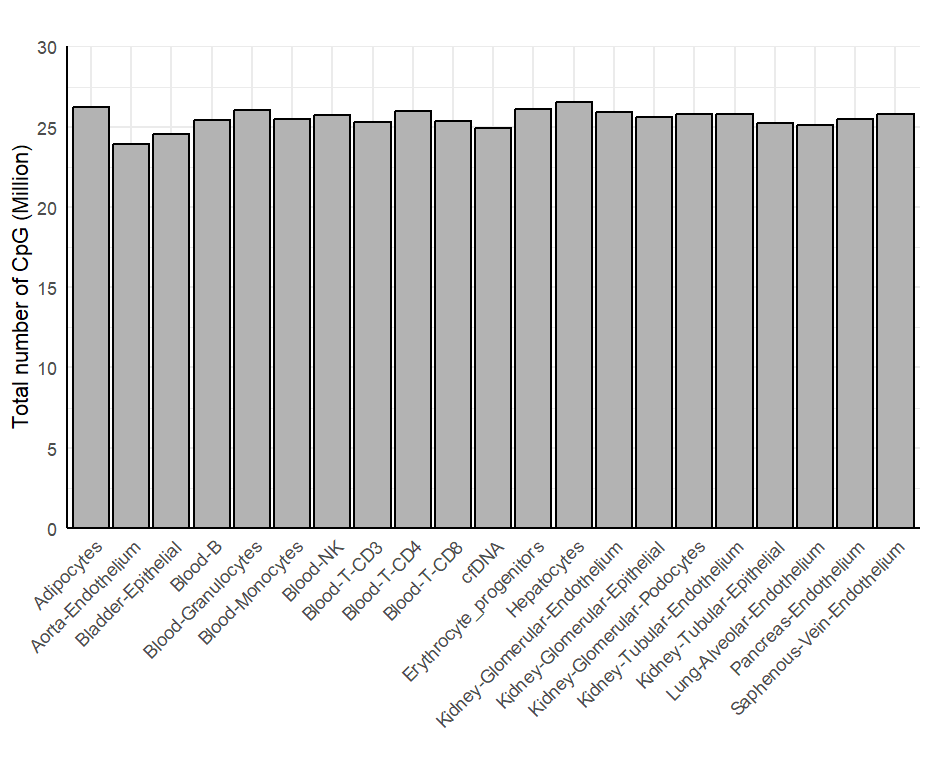
**

**Supplementary Figure 1 :** CpG coverage in core collection of WGBS data from 21 tissues and cell types from GEO (GSE186458).

**
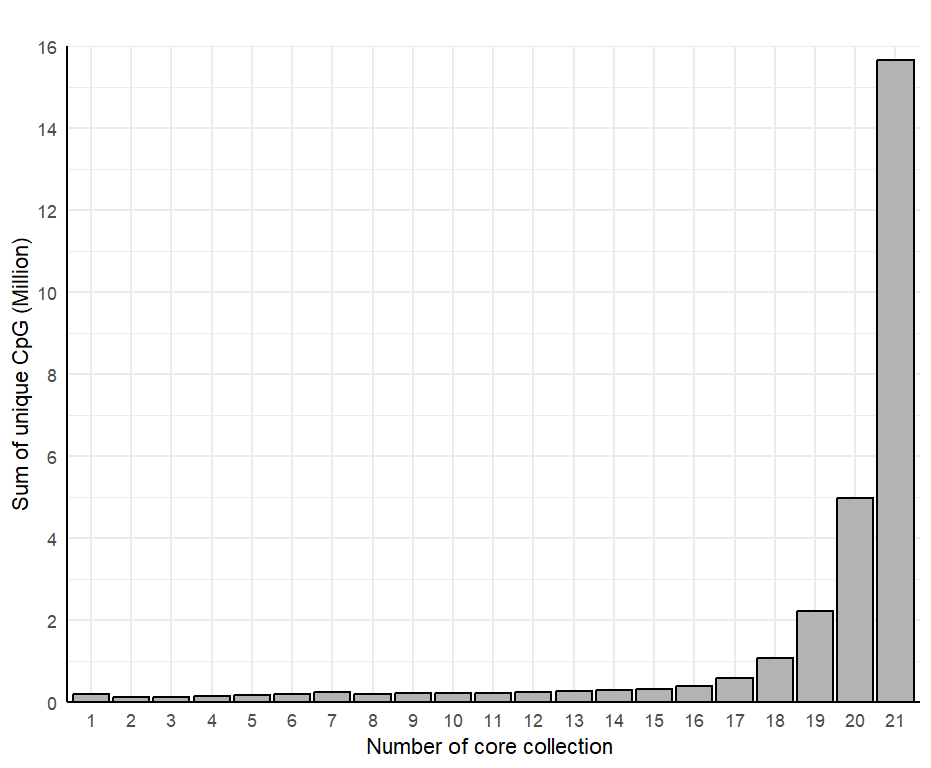
**

**Supplementary Figure 2** : **CpG redundancy in core collection of WGBS data from 21 tissues and cell types from GEO (GSE186458)**. All the 21 core collection are analyzed : the sum of unique CpG per each combination of a given number of core collection is indicated.


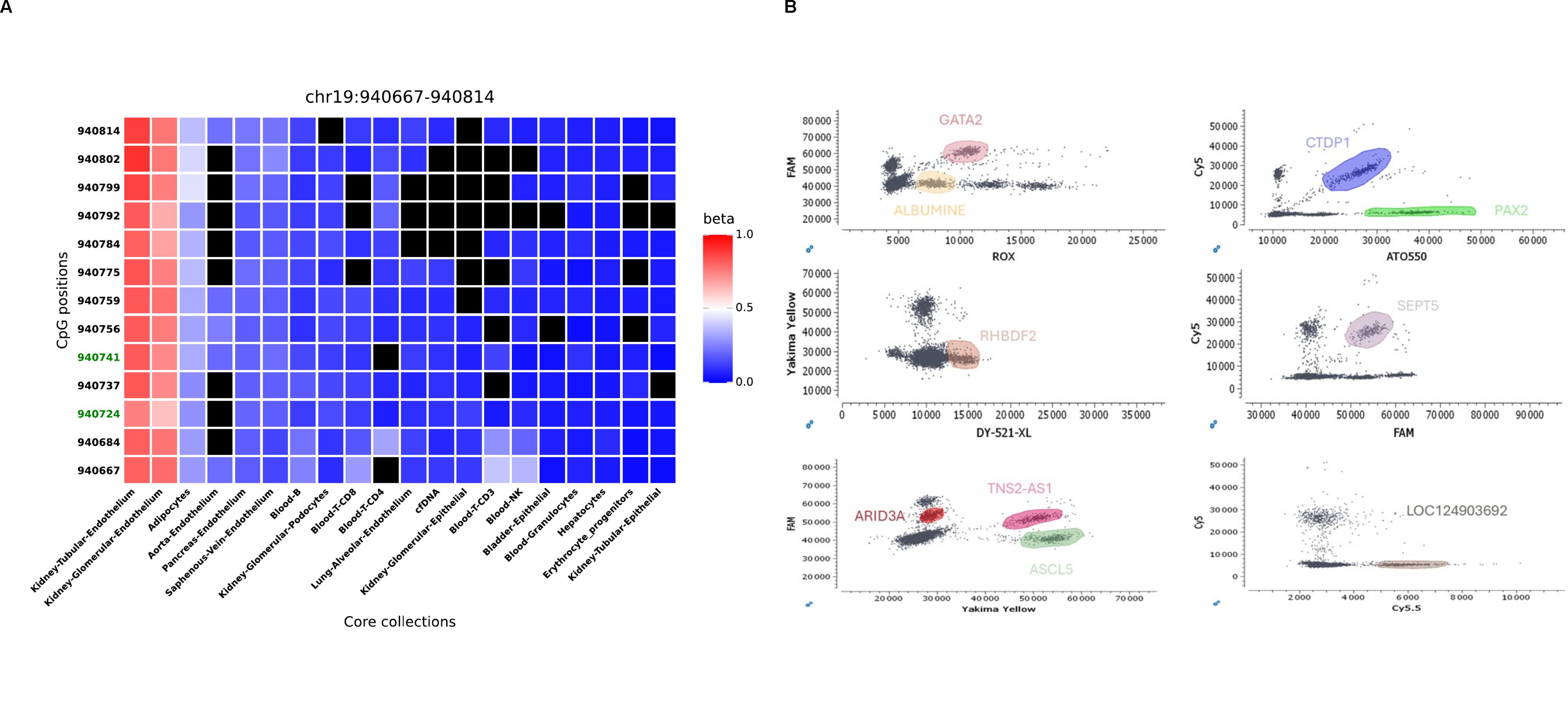


**Supplementary Figure 3: [A] Example of bioinformatic pipeline output and [B] 2D plots of the dPCR assay.** [A] Methylation heatmap of “ARID3A” CpG island with methylation β value (ratio of methylation (0=unmethylated and 1=fully methylated)). Red boxes indicate hypermethylated CpG, blue boxes indicate hypomethylated CpG, black boxes indicate absence of data. Green CpG annotations indicates CpG most differentially methylated between renal cell target considered and other cells included in the *in silico* pipeline. [B] 2D plots of the 10-plex digital-PCR assay on 100% methylated DNA in different detection channels. “GATA2” and “Albumin” internal control are respectively present on the Blue (FAM) and Yellow (ROX) channels ; “CTDP1” and “PAX2” are respectively present on the red (Cy5) and green (ATTO550) channels ; “RHBDF2” is detected in channel long shift (DY-521-XL) ; “SEPT5-GP1BB” is detected on the red (Cy5) and Blue (FAM) channels ; “ARID3A”, “TNS2-AS1” and “ASCL5” are respectively present on Blue (FAM) and Cyan (Yakima Yellow) channels ; “LOC124903692” is detected in the infra-red (Cy5.5) channel.

**Supplementary Table 1:** Heatmaps of methylation β value (ratio of methylation (0=unmethylated and 1=fully methylated)) of the 9 biomarkers “ARID3A”, “GATA2”, “LOC124903692”, “RHBDF2”, “SEPT5-GP1BB”, “TNS2-AS1, “PAX2”, “ACSL5” and “CTDP1”. Green CpG annotations indicates CpG most differentially methylated between renal cell target considered and other cells included in the in silico pipeline.

| Biomarkers name | Renal cell(s) target | Heatmap | Chromosomal position CpG Islands | Nucleotide position of CpG islands on the gene |
| --- | --- | --- | --- | --- |
| ARID3A | Vascular renal cells | 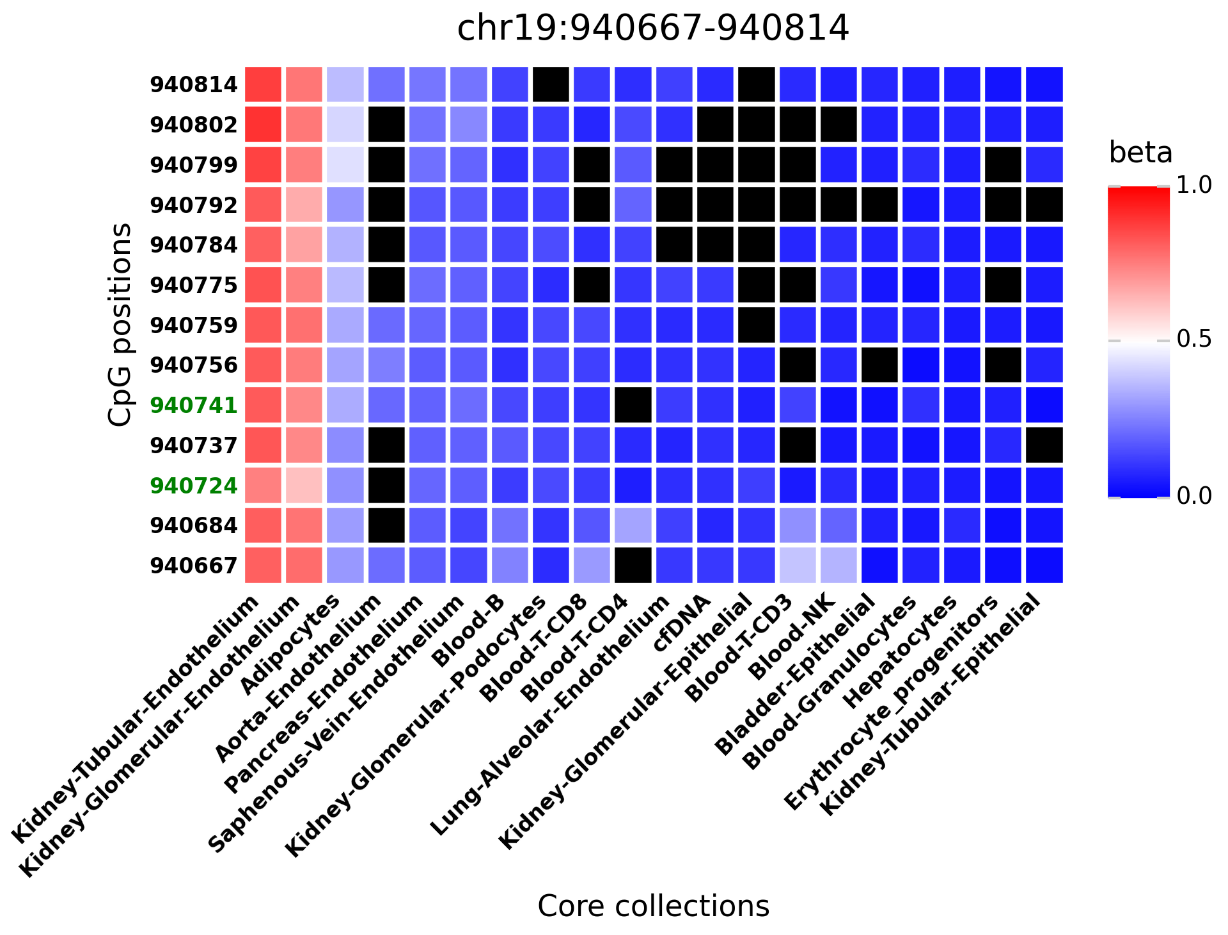 | chr19 | 940,667-941,160 |
| ACSL5 | Epithelial renal cells | 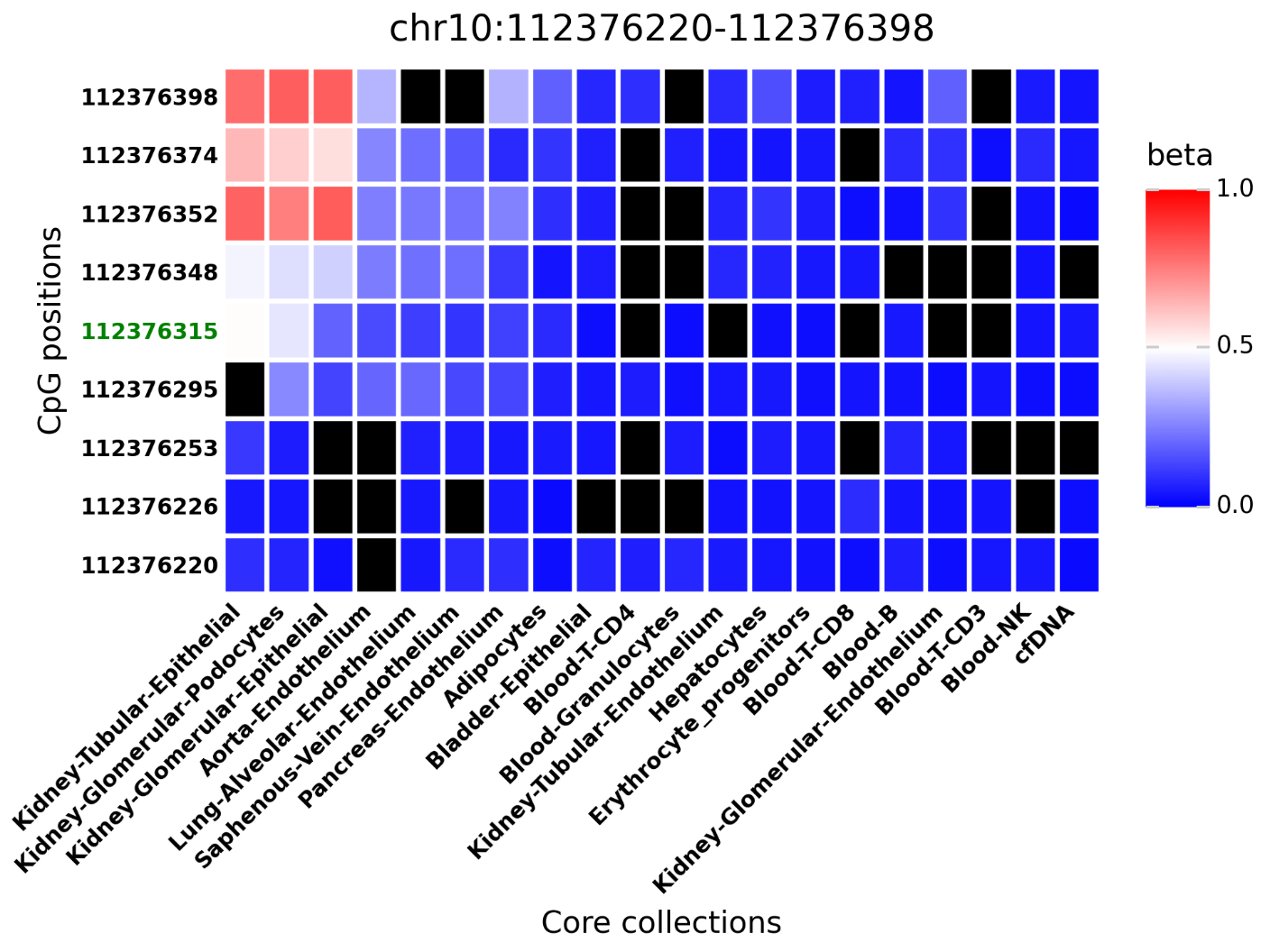 | 10 | 112,376,275-112,376,398 |
| CTDP1 | Pan-kidney cells | 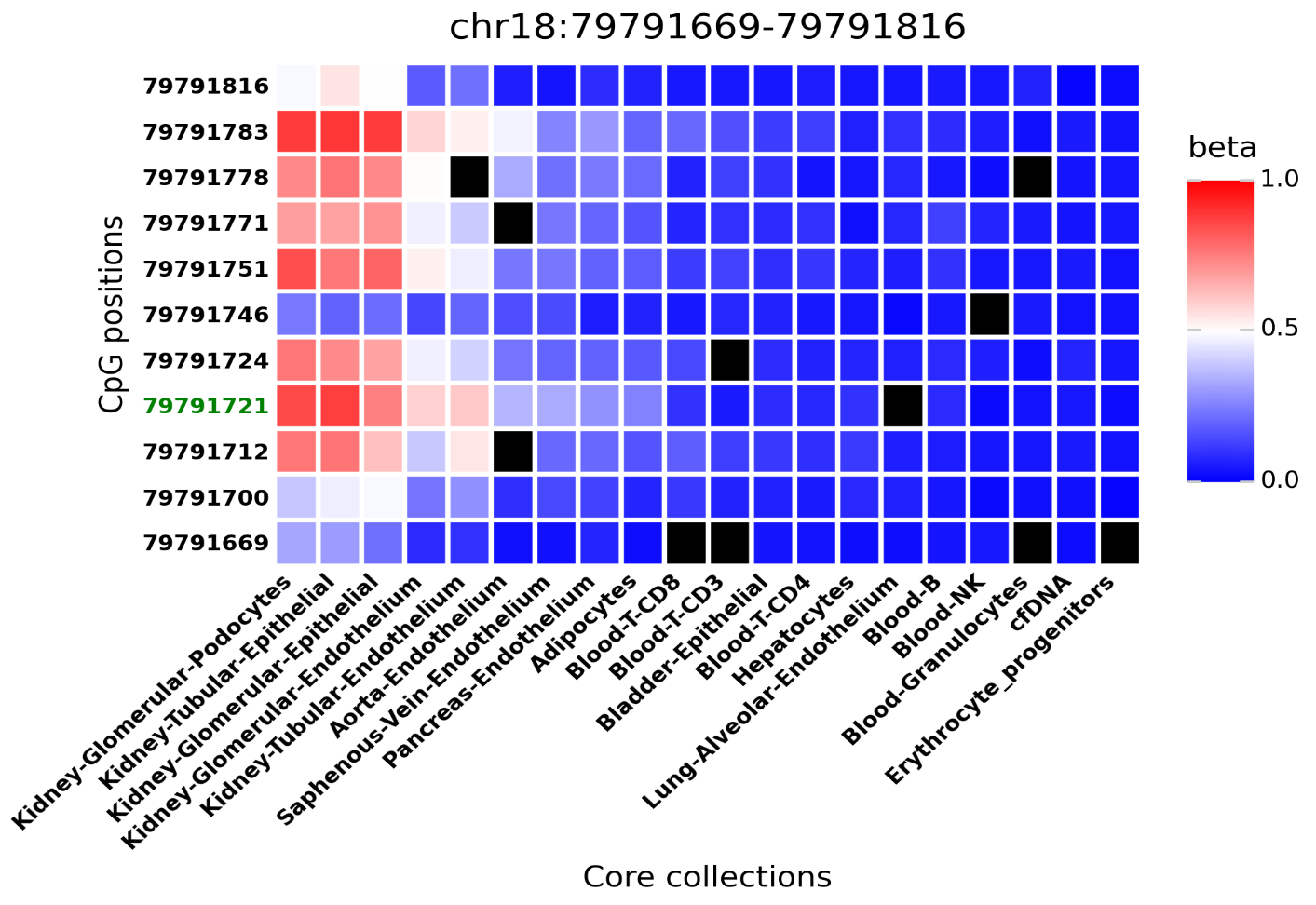 | 18 | 79,791,700-79,791,820 |
| GATA2 | Vascular renal cells | 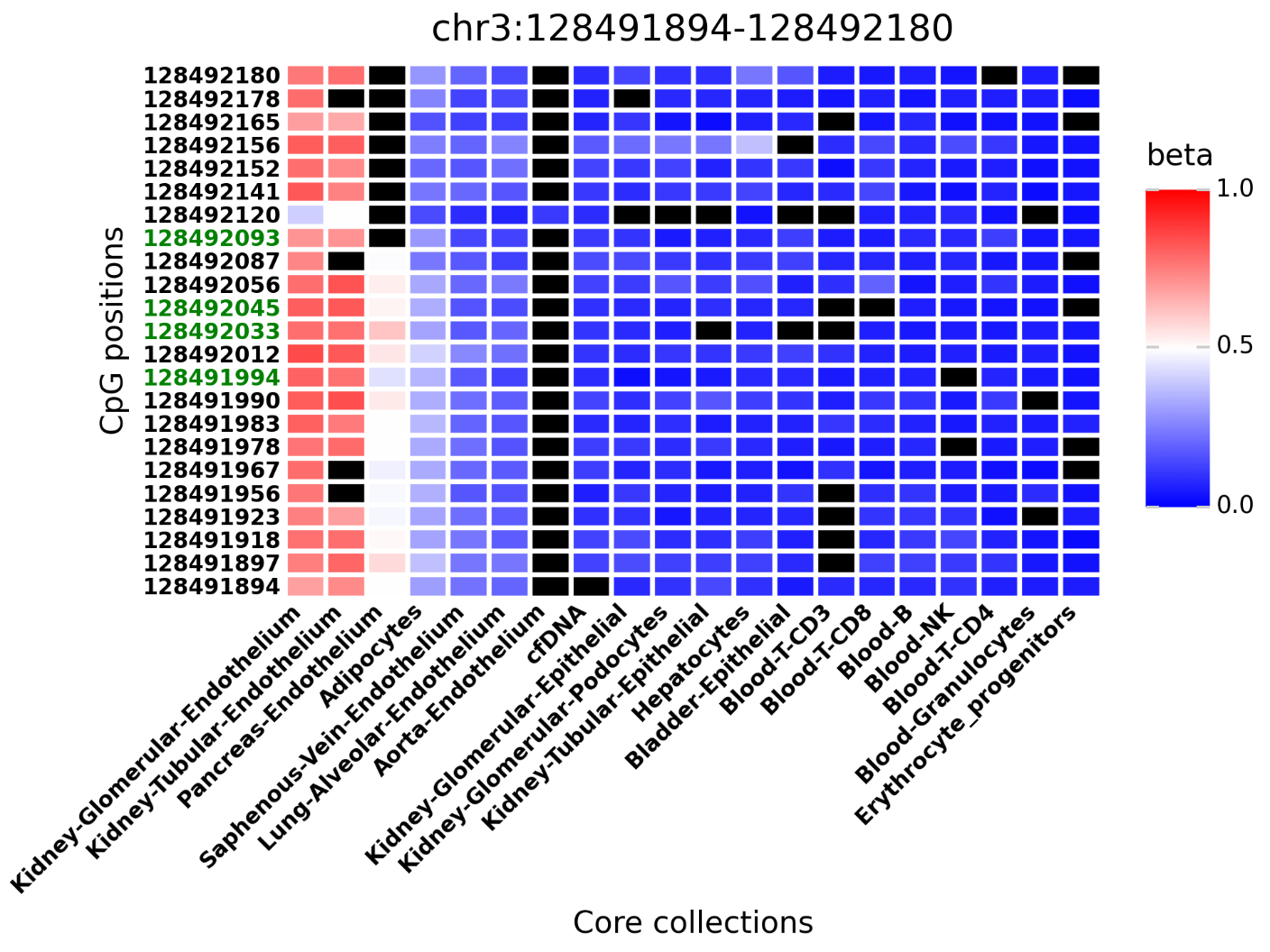 | 3 | 128,491,794-128,492,245 |
| LOC124903692 | Vascular renal cells | 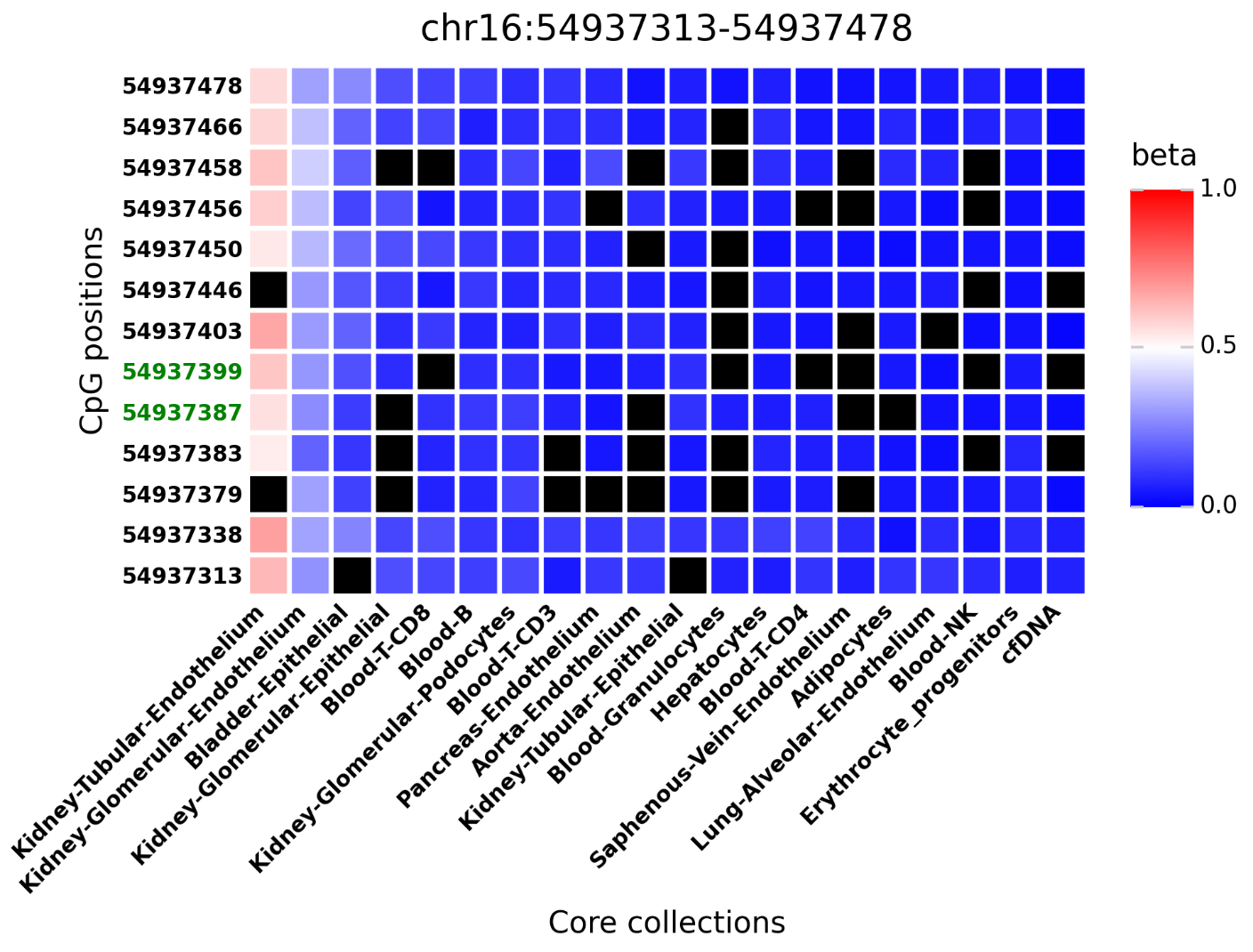 | 16 | 54,937,300-54,937,500 |
| PAX2 | Epithelial renal cells | 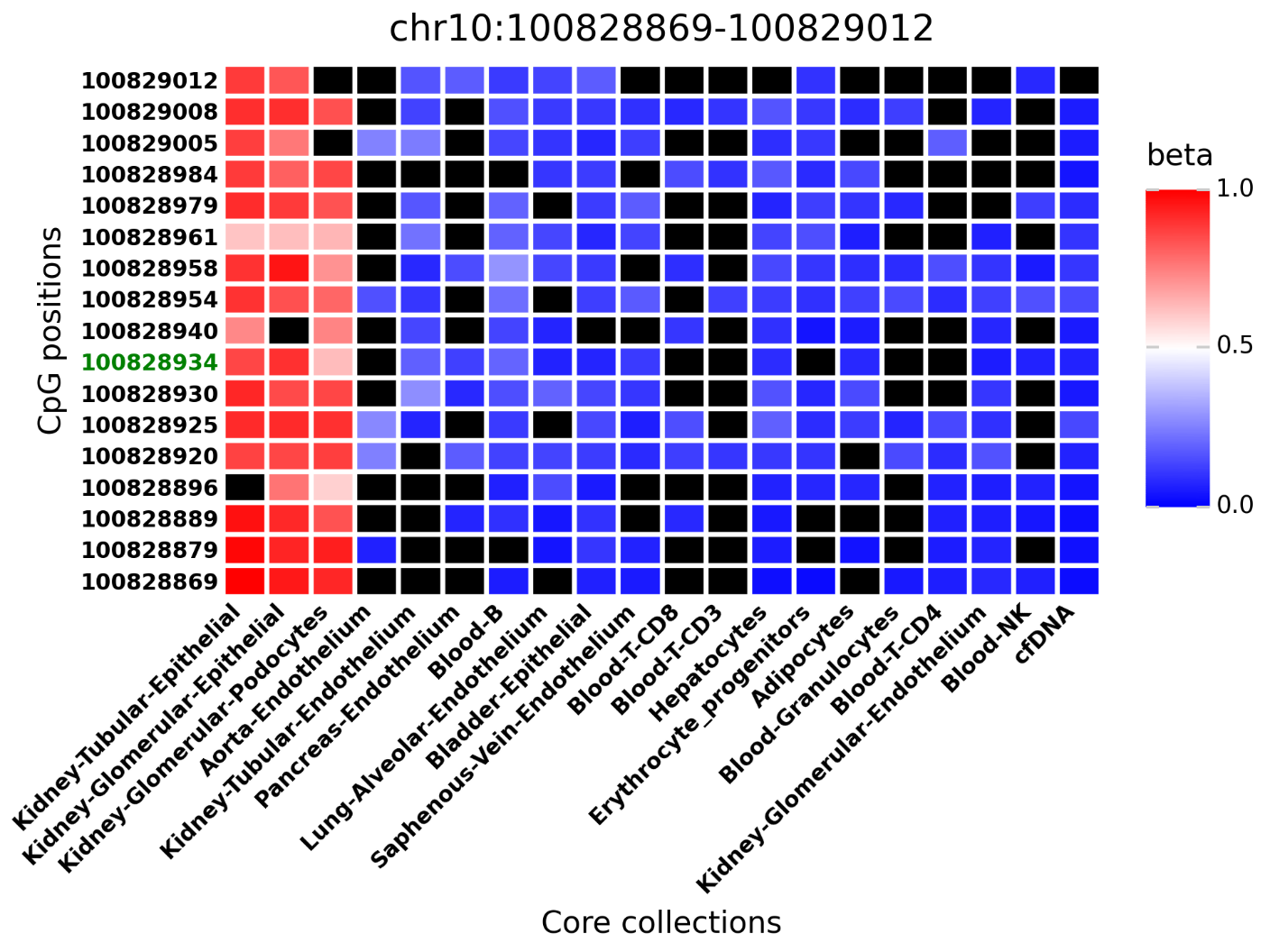 | 10 | 100,828,800-100,829,020 |
| SEPT5-GP1BB | Vascular renal cells | 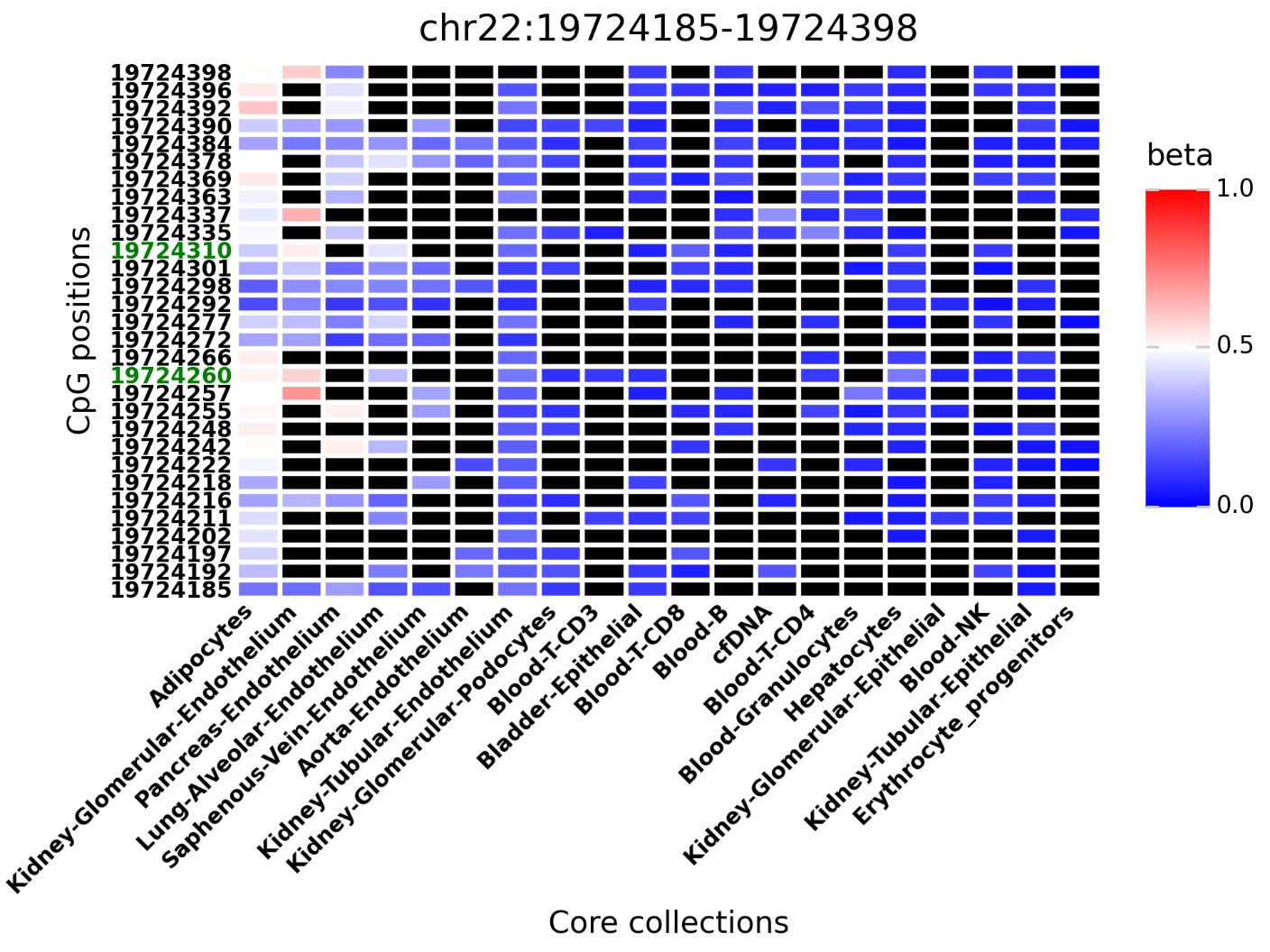 | 22 | 19,724,235-19,724,340 |
| TNS2-AS1 | Vascular renal cells | 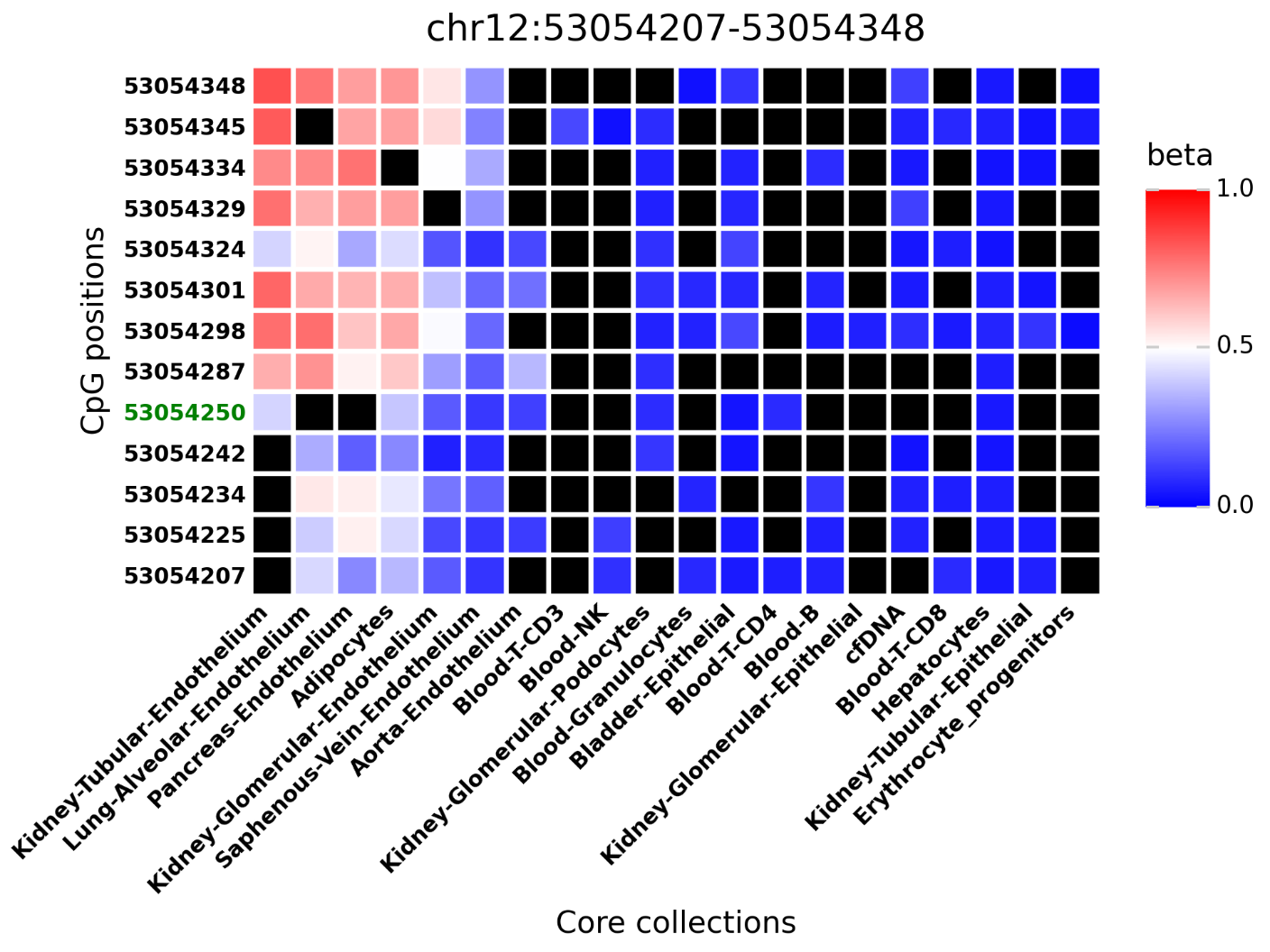 | 12 | 53,054,207-53,054,329 |
| RHBDF2 | Vascular renal cells | 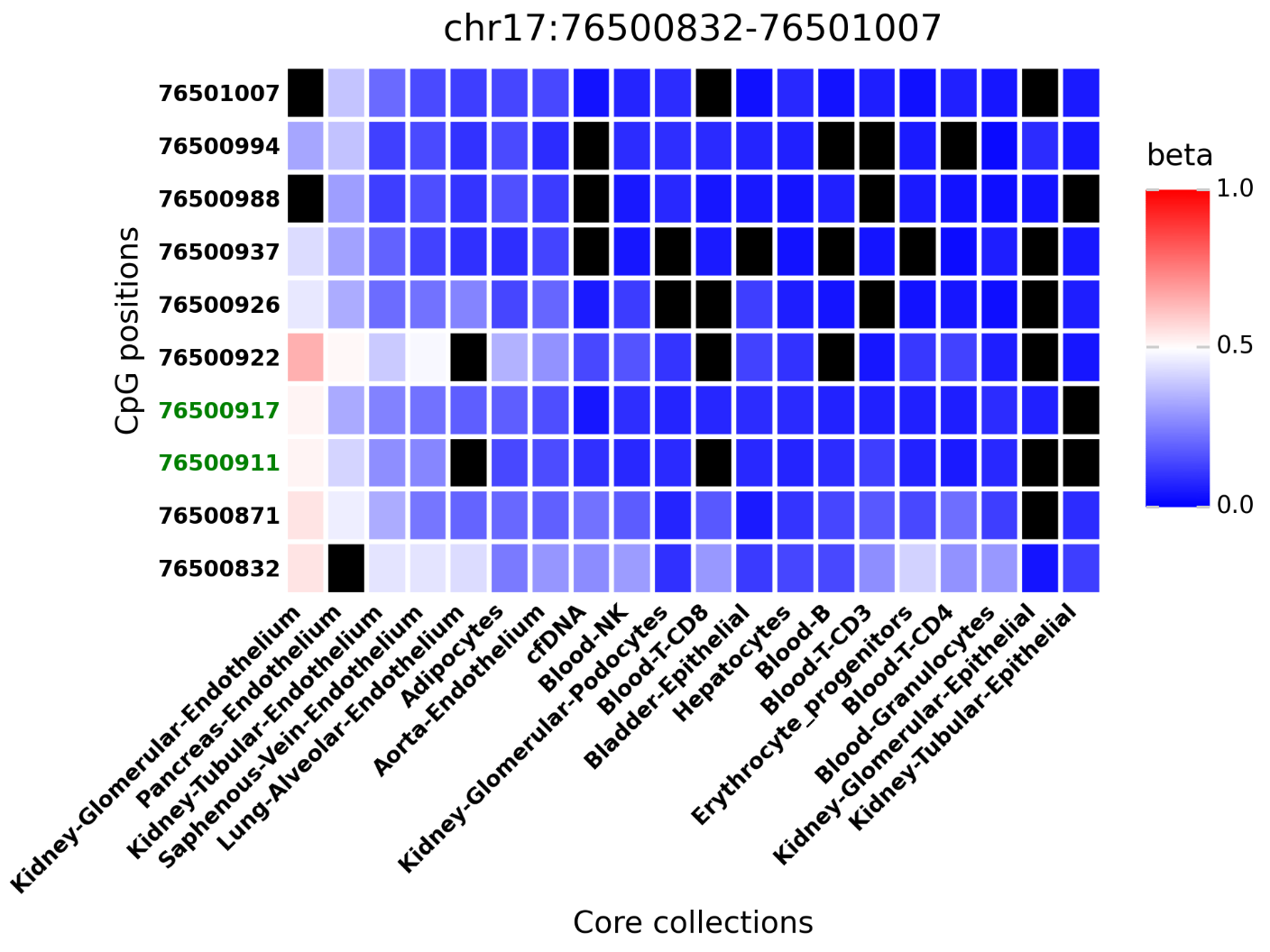 | 17 | 76,470,893-76,501,427 |

| **Target** | **Manufacturer** | **Host** | **Fluorochrome** | **Reference** | **Clone** | **Laser** | **BP Filter** |
| --- | --- | --- | --- | --- | --- | --- | --- |
| **CD105** | BD | Mouse | BUV395 | 563803 | 266 | 355nm | 379/28 |
| **EpCAM**  **(CD326)** | Biolegend | Mouse | APC | 324208 | 9C4 | 633nm | 670/14 |
| **CD45** | Biolegend | Mouse | APC | 368512 | 2D1 | 633nm | 670/14 |
| **CD235ab** | Biolegend | Mouse | APC | 306608 | HIR2 | 633nm | 670/14 |
| **Near-IR**  **Viability Dye** | Thermofisher | / | / | L34975 | / | 633nm | 780/60 |

**Supplementary Table 2: List of antibodies used for kidney endothelial cells sorting**

| **Target** | **Manufacturer** | **Host** | **Fluorochrome** | **Reference** | **Clone** | **Laser** | **BP Filter** |
| --- | --- | --- | --- | --- | --- | --- | --- |
| **CD105** | BD | Mouse | APC | 562408 | 266 | 633nm | 670/14 |
| **EpCAM**  **(CD326)** | Biolegend | Mouse | BUV737 | 748382 | KS1/4 | 355nm | 740/35 |
| **CD45** | Biolegend | Mouse | APC | 368512 | 2D1 | 633nm | 670/14 |
| **CD235ab** | Biolegend | Mouse | APC | 306608 | HIR2 | 633nm | 670/14 |
| **Near-IR**  **Viability Dye** | Thermofisher | / | / | L34975 | / | 633nm | 780/60 |

**Supplementary Table 3: List of antibodies used for kidney epithelial cells sorting**


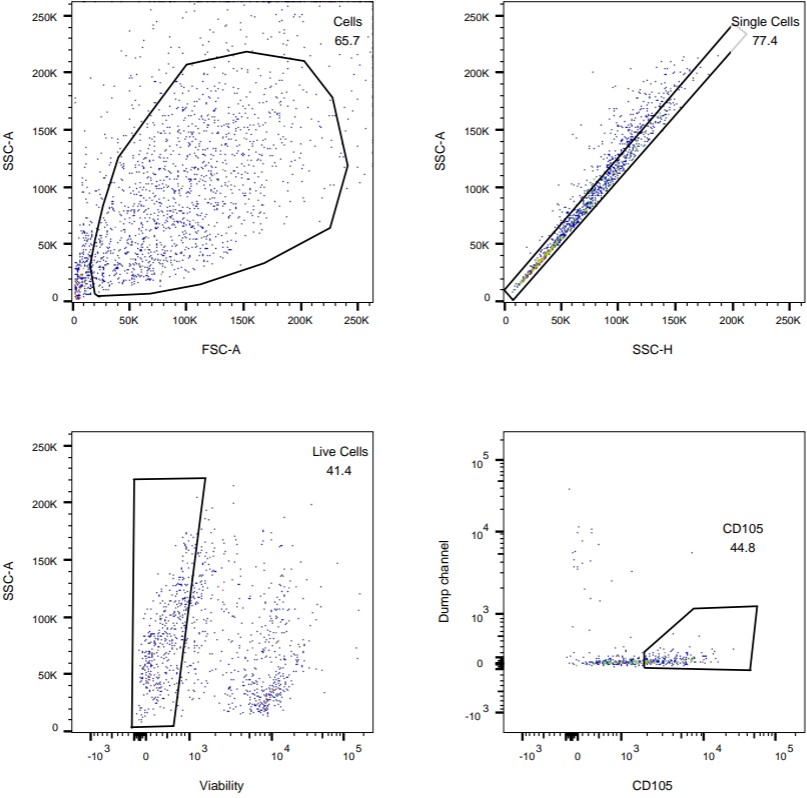
**Supplementary Figure 4 : Flow cytometry gating strategy for sorting endothelial kidney cell in the tubular fraction.** Cell gate => Single Cells => Live cells (not stained by the viability marker) => Endothelial cells (CD105+).

**
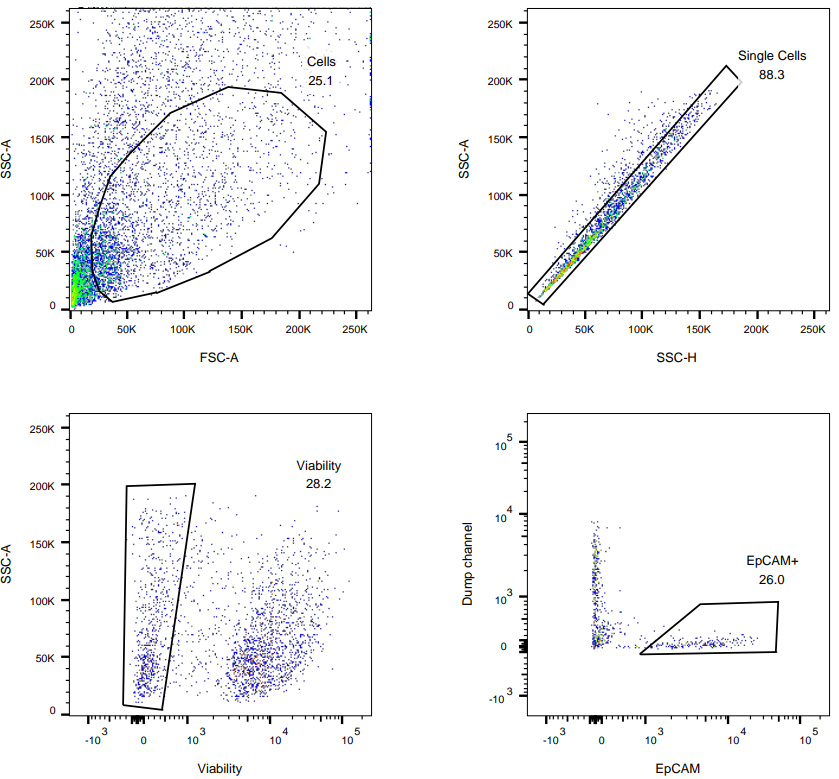
**

**Supplementary Figure 5: Flow cytometry gating strategy for sorting epithelial kidney cells in the tubular fraction.** Cell gate => Single Cells => Live cells (not stained by the viability marker) => Epithelial cells (EpCAM+).


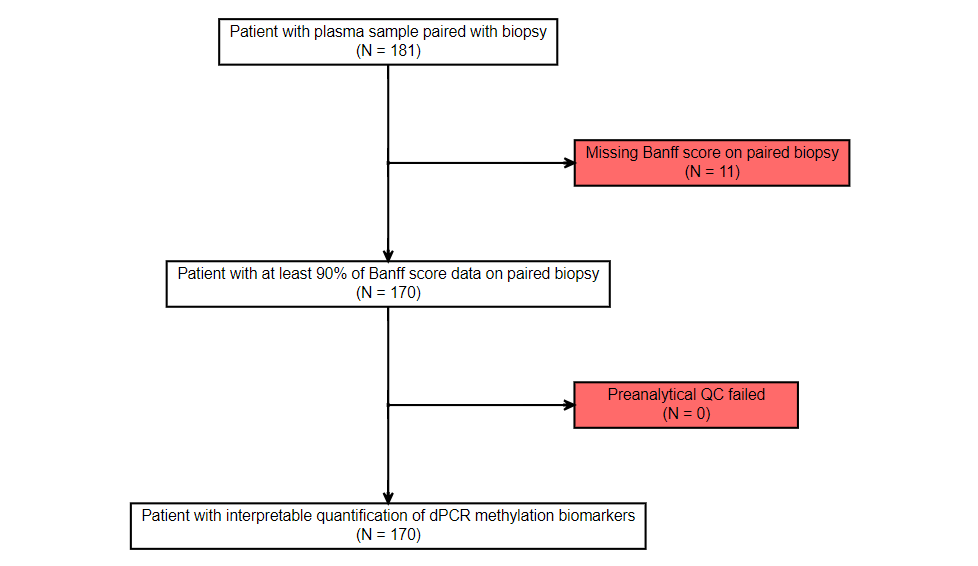


**Supplementary Figure 6 : Flowchart of plasma sample selection for statistical analysis in the multicentric cohort of kidney transplant patient.**  11 samples were excluded due to missing Banff score on paired biopsy.

**Supplementary Table 4 : Clinical characteristics of the multicentric cohort of KTRs with paired plasma sample and solid biopsy.** ABMR : Antibody-Mediated Rejection; TCMR : T-cell Mediated Rejection; SD : Standard Deviation.

| Characteristics | N | N = 170^1^ |
| --- | --- | --- |
| Age | 169 |  |
| Mean (SD) |  | 56.3 (14.6) |
| Median (Range) |  | 57.0 (22.5, 89.0) |
| Missing |  | 1 |
| Sex | 169 |  |
| Female |  | 70 (41%) |
| M |  | 99 (59%) |
| Missing |  | 1 |
| Type of biopsy | 165 |  |
| Cause |  | 89 (54%) |
| Protocolar |  | 76 (46%) |
| Missing |  | 5 |
| Rejection | 170 |  |
| No |  | 126 (74%) |
| Yes |  | 44 (26%) |
| Rejection Type | 168 |  |
| ABMR |  | 24 (14%) |
| Mixed |  | 7 (4.2%) |
| No rejection |  | 124 (74%) |
| TCMR |  | 13 (7.7%) |
| Missing |  | 2 |
| C4 deposit | 164 |  |
| Absence |  | 146 (89%) |
| Presence |  | 18 (11%) |
| Missing |  | 6 |
| Donor-specific antibodies | 169 |  |
| Absence |  | 120 (71%) |
| Presence |  | 49 (29%) |
| Missing |  | 1 |
| eGFR (mL/min/1.73m2) | 160 |  |
| Mean (SD) |  | 42.8 (22.9) |
| Median (Range) |  | 39.5 (0.0, 113.0) |
| Missing |  | 10 |
| ^1^n (%) | | |

| Biomarkers | Sens Primers | Reverse Primers | Probes | |
| --- | --- | --- | --- | --- |
| Name | Sequence | Sequence | Sequence | Fluorochrome |
| ARID3A | GCGGTTGTGTTTGAGATAGAG | CAAAACTAACGACCGAAAACC | TGTTAAGAGAGTTTCGAGT | FAM |
| ACSL5 | GGATATTTTGGGTCGGTTTTTTGT  TGGGTCGGTTTTTTGTTTGTATG | CTCGCTCGTAATTCCTCCAAA  TCCCTCGCTCGTAATTCCTC | TG{T}ATG{G}ACG{T}TTTG{A}AG{T}T | YY |
| CTDP1 | GAAACGGTGAGATGTTCGGAG GGTTTAAATGAAACGGTGAGATGT | CTTTACTATTACGAAACGTTCCTAATAC ACGAAACGTTCCTAATACAACAA | AA{G}CG{T}CGGTTG{T}TTGT | Cy5/ATTO550 |
| GATA2 | TTGGTTTTCGAATTTTGTTTGTTTATA | CTAATAATAAACTCCAAATCTACGCA | ACGA{A}TTT{T}AGT{A}CGGG{G}T | FAM/ROX |
| LOC124903692 | TGGGGGAGGGAAGGTCG | CTATAAAAAACAAATAAACTAAAAAACACG ACAAATAAACTAAAAAACACGATCGA | TGCGGTTTTGGTATCGA ACGTGCGGTTTTGGT | Cy5.5 |
| PAX2 | GGCGAAATTCGGTGTATATAATTT | GAACGACAAAAACTCTACGAAA | TC{G}TTTC{G}TTTC{G}TTCG | ATTO550 |
| SEPT5-GP1BB | CGTTTGTTGTTTTATTTGGTCGA  TGGTCGAGGACGAGTTGC | ACAAAACGAACCGAAAACGC  CGCGTACAACAACCCAAAA | ACG{A}GTT{G}CGCGTCGT | Cy5/FAM |
| TNS2-AS1 | AGTTATTTTCGGGTGTTTCGTT  CGGGTGTTTCGTTTTTGCG | CGAATCCAAACTAAAACTCCTAAAA  CGCTCCATAAACGAATCCAAAC | TGCGT{T}TAT{G}CGTAG{G}TT | FAM/YY |
| RHBDF2 | TCGTATTTGTTTTTATTTTGGTTGGT | CTTTCTCCCGAATAAACACCG | TAAA{T}TTCGGTTTCG{G}TTCGT | DY-521-XL |
| Albumin  Internal control | GGGATGGAAAGAATTTTATGTT | AGAATTTGGGGTTAGTTTGTTT | AGGGTTTTTATAATTTA | ROX |

Probes and primers are provided individually by Eurogentec and are validated for *in vitro* diagnostic testing. [C]; [T] ; [A] ; [G] composing the Taqman probes are “LNA modified nucleotides” to increase the melting PCR temperature and fit with the hybridization temperature of the primer.

**Supplementary Table 5: Probes and Primers sequences description for the set of 9 biomarkers (including “Albumin” sequence as internal control).**

| **PCR reagents** | **Initial Concentration in Reactional Mix (µM)** |
| --- | --- |
|  |  |
| Primer FORWARD **ALB** | 0.7 |
| Primer REVERSE **ALB** | 0.7 |
| PROBE **ALB** - ROX | 0.6 |
| Primer FORWARD **ARID3A** | 0.6 |
| Primer REVERSE **ARID3A** | 0.6 |
| PROBE **ARID3A** (FAM) | 0.6 |
| Primer FORWARD **TNS2-AS1** | 0.55 |
| Primer REVERSE **TNS2-AS1** | 0.55 |
| PROBE **TNS2-AS1** (FAM) | 0.55 |
| PROBE **TNS2-AS1** (YY) | 0.55 |
| Primer FORWARD **GATA2** | 0.6 |
| Primer REVERSE **GATA2** | 0.6 |
| PROBE **GATA2** (FAM) | 0.4 |
| PROBE **GATA2** (ROX) | 0.4 |
| Primer FORWARD **PAX2** | 0.5 |
| Primer REVERSE **PAX2** | 0.5 |
| PROBE **PAX2** (ATTO550) | 0.45 |
| Primer FORWARD **CTDP1** | 0.55 |
| Primer REVERSE **CTDP1** | 0.55 |
| PROBE **CTDP1** (ATTO550) | 0.4 |
| PROBE **CTDP1** (Cy5) | 0.4 |
| Primer FORWARD **ACSL5** | 0.65 |
| Primer REVERSE **ACSL5** | 0.65 |
| PROBE **ACSL5** (YY) | 0.5 |
| Primer FORWARD **LOC124903692** | 0.6 |
| Primer REVERSE **LOC124903692** | 0.6 |
| PROBE **LOC124903692** (Cy5.5) | 0.5 |
| Primer FORWARD **RHBDF2** | 1.5 |
| Primer REVERSE **RHBDF2** | 1.5 |
| PROBE **RHBDF2** (DY-521-XL) | 1.25 |
| Primer FORWARD **Sept5** | 0.5 |
| Primer REVERSE **Sept5** | 0.5 |
| PROBE **Sept5** (FAM) | 0.4 |
| PROBE **Sept5** (Cy5) | 0.4 |

**Supplementary Table 6 : Detailed description Assay Mix containing mix of primers & probes.**


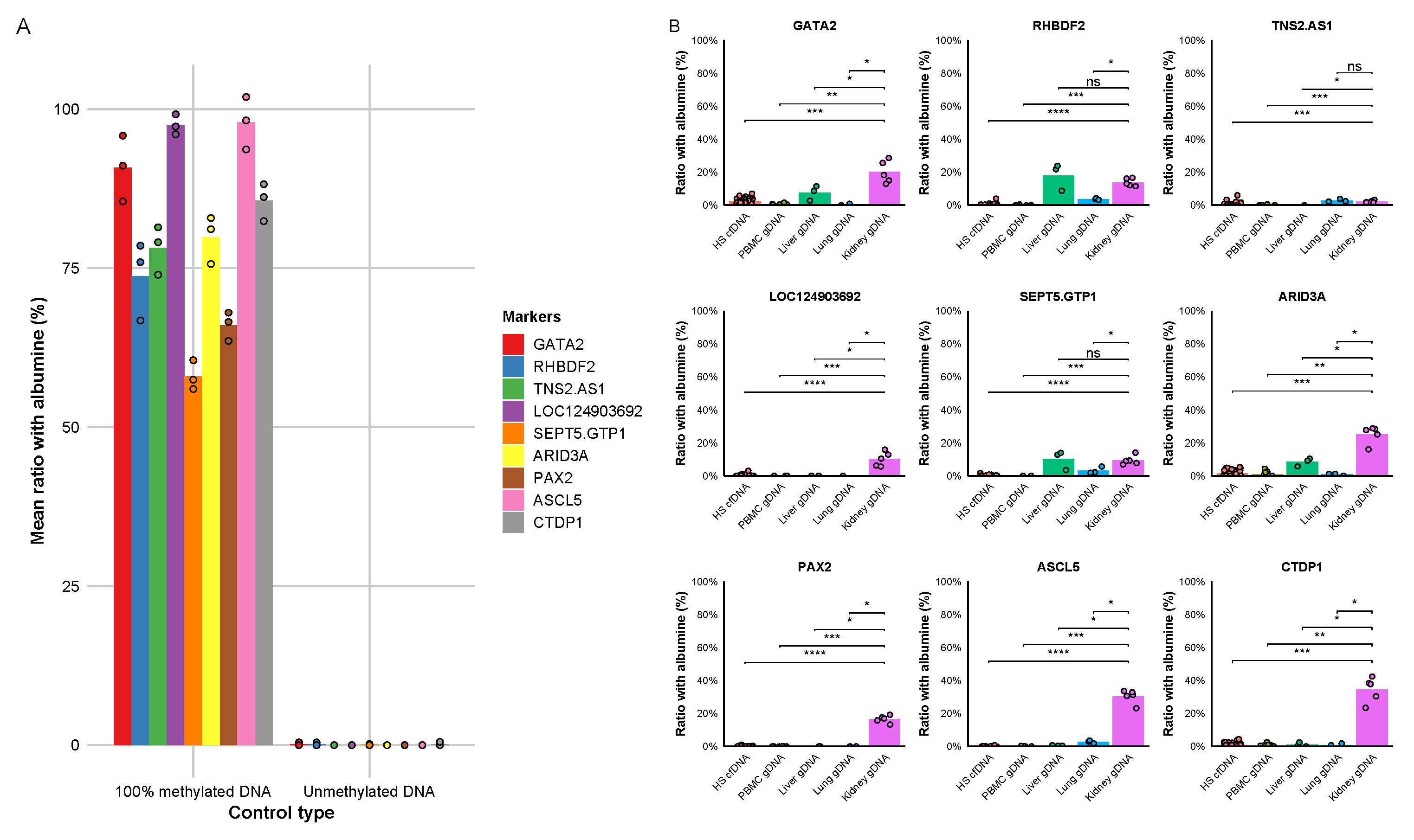


**Supplementary Figure 7: Relative quantification of each epigenetic kidney biomarker in synthetic control.** Copy number of biomarkers were normalized by the copy number of genomes represented by “Albumin”. Barplots show mean value of n=3 sample. Individual quantifications are shown by dots.

| **Characteristic** | **N** | **N = 170** |
| --- | --- | --- |
| **Albumin** | 170 |  |
| Mean (SD) |  | 7,330.8 (19,810.9) |
| Median (Range) |  | 2,363.8 (137.5, 229,900.0) |
| **GATA2** | 170 |  |
| Mean (SD) |  | 65.2 (119.9) |
| Median (Range) |  | 37.9 (0.0, 1,345.3) |
| **CTDP1** | 170 |  |
| Mean (SD) |  | 24.3 (57.7) |
| Median (Range) |  | 10.6 (0.0, 612.1) |
| **PAX2** | 170 |  |
| Mean (SD) |  | 0.26 (1.5) |
| Median (Range) |  | 0.0 (0.0, 15.1) |
| **RHBDF2** | 170 |  |
| Mean (SD) |  | 4.0 (9.0) |
| Median (Range) |  | 0.0 (0.0, 48.5) |
| **ASCL5** | 170 |  |
| Mean (SD) |  | 1.3 (3.9) |
| Median (Range) |  | 0.0 (0.0, 31.1) |
| **TNS2.AS1** | 170 |  |
| Mean (SD) |  | 8.9 (20.2) |
| Median (Range) |  | 0.0 (0.0, 176.0) |
| **LOC124903692** | 170 |  |
| Mean (SD) |  | 16.5 (75.0) |
| Median (Range) |  | 3.0 (0.0, 929.5) |
| **SEPT5.GTP1** | 170 |  |
| Mean (SD) |  | 5.8 (39.4) |
| Median (Range) |  | 0.0 (0.0, 506.8) |
| **ARID3A** | 170 |  |
| Mean (SD) |  | 63.6 (140.7) |
| Median (Range) |  | 31.3 (0.0, 1,300.6) |

**Supplementary Table 7: Mean quantification of methylation biomarker candidate and “Albumin” total genome (cp/mL of sample) in the multicentric cohort of N=170 plasma sample from KTRs patients.** SD : Standard Deviation.

**A
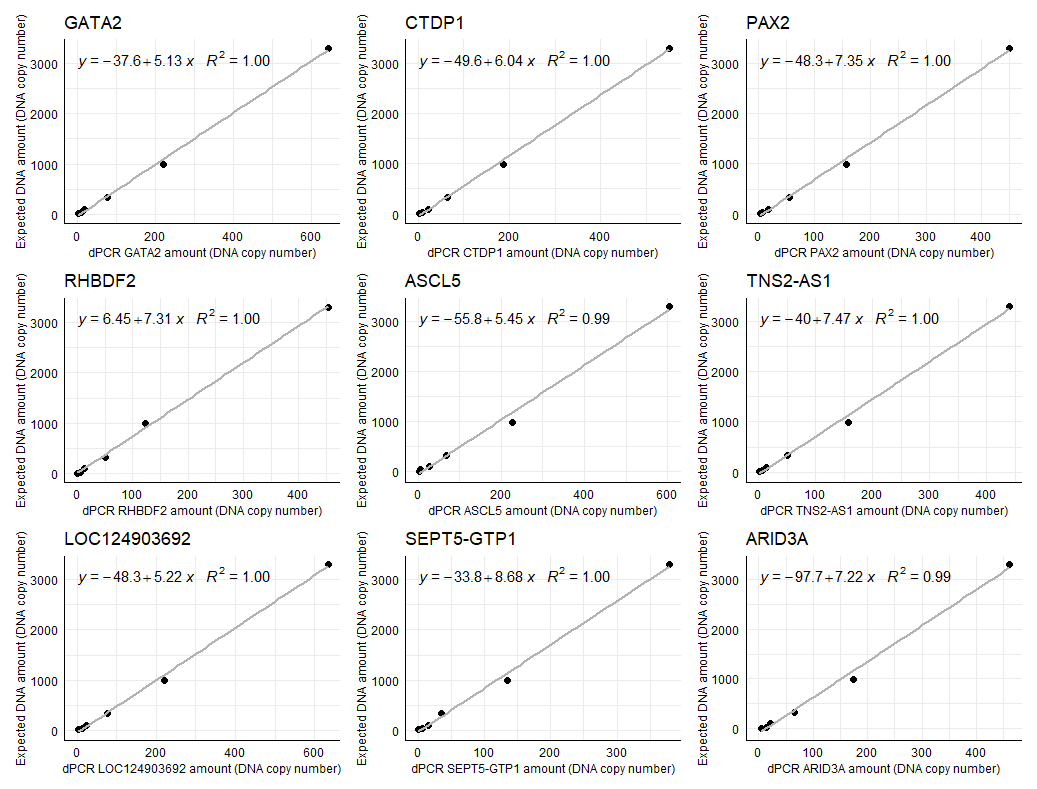
**

**B**

**
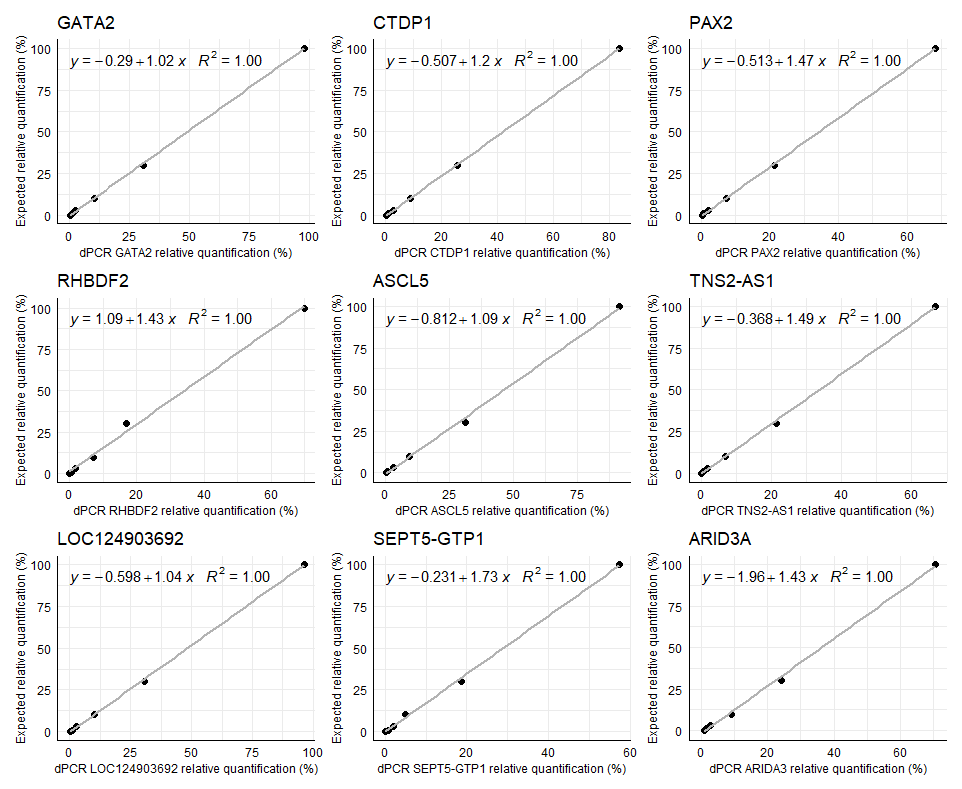
**

**Supplementary Figure 8**: Analytical sensitivity for each biomarker of the 10-plex digital-PCR assay [A] in DNA copy number and [B] relative quantification to “Albumin”. Y-axis shows the expected value of quantification for the 6 control samples used (see “Analytical Sensitivity” section of the Material and Methods) while X-axis shows the values of quantification obtained after bisulfite convertion and dPCR analysis of the 6 control samples.
